# Exclusive breastfeeding practice among HIV infected mothers in the southern highlands of Tanzania; assessing the prevalence and factors associated with the practice, an analytical cross-sectional survey

**DOI:** 10.1186/s12981-022-00451-6

**Published:** 2022-06-27

**Authors:** Rose Faustine, Fabiola Vincent Moshi

**Affiliations:** 1 Department of Clinical Nursing, School of Nursing and Public Health, The University of Dodoma, P.O. Box 259, Dodoma, Tanzania; 2 Department of Nursing Management and Education, School of Nursing and Public, Health of University of Dodoma, P.O. Box 259, Dodoma, Tanzania

**Keywords:** Exclusive breastfeeding, HIV exposed infants, And HIV, Mothers, Tanzania

## Abstract

**Background:**

There is no other better way to safeguard an infant’s health in the first 6 months of life than exclusive breastfeeding (EBF). Breast milk is valuable in all aspects of an infant’s physical and mental growth as well as immune development. The study aimed to assess the prevalence and factors associated with EBF practice among HIV-infected mothers in the Southern Highlands of Tanzania.

**Method:**

A hospital-based analytical cross-sectional study was conducted among lactating HIV-infected mothers. A random sampling procedure was used to obtain 372 HIV-infected mothers of infants from 6 to 12 months of age who were still breastfeeding at the time of data collection. An interviewer-administered structured questionnaire was used for data collection. Bivariate and multivariable logistic regression was used to assess factors associated with EBF practice. Statistical package for social science (SPSS volume 20) software was used for data entry and analysis.

**Results:**

The prevalence of EBF practice was 58.1% at 95% Confidence Interval of 52.9% to 63.1%. More than half of the respondents 199 (53.5%) had adequate knowledge while 173(46.5%) had inadequate knowledge about EBF. After adjusting for confounders, factors associated with EBF practice were knowledge about EBF [Adequate knowledge (AOR = 5.11 at 95% CI 3.2–8.17, p < 0.001)], ANC visits [Adequate (AOR = 1.76 at 95% CI 1.09–2.82, p = 0.002)], Income per day [1 0r more USD (AOR = 1.83 at 95% CI 1.14–2.94, p = 0.013)], positive perception of EBF [ positive perception (AOR = 3.51 at 95% CI 2.25–5.47, p < 0.001) and having ever experienced a breast problem AOR = 3.91 at 95% CI 1.89–8.08, p < 0.001.

**Conclusion:**

More than half of interviewed mothers with HIV practiced EBF. The EBF practice among HIV lactating mothers was significantly influenced by adequate knowledge of EBF, positive perception toward EBF, adequate ANC visits, and having never experienced breast problems. Strengthening adherence to ANC routine visits, counseling on breastfeeding, and improving mothers’ knowledge about exclusive breastfeeding would contribute to the enhancement of EBF practice in this region. An innovative interventional study is recommended to develop more effective strategies to improve EBF knowledge and practice among HIV-infected mothers.

## Background

Human immunodeficiency virus (HIV) is a virus that attacks the immune system of an infected person and exposes the body to several opportunistic infections [1]. A major means of HIV transmission is unprotected sex, but a significant majority of transmission still occurs from mother to child [2]. Mother-to-child transmission happens when HIV is transmitted from the mother to the child during pregnancy, during birth, or while breastfeeding [1]. Exclusive breastfeeding (EBF) is the process in which the infant gets only breast milk for the first 6 months of life and nothing else, except for ORS, minerals, vitamins, and medicines [3].

Screening for HIV during pregnancy and promotion of EBF for all neonates is a key strategy for the prevention of HIV infection from mother to child. Screening for HIV infection during pregnancy provides an opportunity for early diagnosis of HIV infection and timely initiation of antiretroviral drugs [4]. All pregnant women need to be screened for HIV infection because the prevalence and incidence of HIV infection are still high. In 2018, it was estimated that about 37.9 million people were HIV positive [5]. Similarly, 1.7 million people became newly infected with HIV in 2018 globally [5]. For the African Region, the WHO observed that in the same year, there was an acute problem whereby there were about 25.7 million people were living with HIV infection and 1.1 million people were newly infected 2018 [6]. It was also estimated that, in 2018, about 1.1 pregnant women were living with HIV infection globally [6]. Pregnant women infected with HIV should take antiretroviral drugs throughout pregnancy and childbirth and post-delivery to prevent mother-to-child transmission of HIV infection [7].

According to WHO guidelines, in settings where people who are living with HIV infection have lifelong ART support, breastfeeding should not be restricted [8]. Infected mothers should breastfeed their infants exclusively for the first 6 months of life, introduce appropriate complementary foods thereafter, and continue breastfeeding. Breastfeeding should only be stopped once a nutritionally adequate and safe diet without breast milk can be provided [8]. Standards of infant feeding in the global north and global south vary due to the availability of nutritionally adequate diets [9]. In the global north, women with HIV do not breastfeed their babies because of the availability of alternative feeding [9].

In the global south, exclusive breastfeeding for 6 months after birth has been demonstrated to lower the risk of vertical transmission of HIV [10]. In sub-Saharan Africa, where the prevalence and incidence of HIV infection are high, the proportion of EBF among all women is reported to be low due to the incompatibility of EBF and African traditional beliefs and cultural practices [11]. It is evidenced that HIV Infected lactating mothers are less likely to practice EBF if compared to non-infected lactating mothers [12]. In Tanzania, EBF is under practiced among women infected with HIV/AIDS (46%) [13] if compared to the general population(59%) [14].

Breastfeeding has a significant function in the nutrition, health, and cognitive growth of infants because human milk is the optimal nutrition for infants’ survival, development, and growth [15]. If infants get well breastfed in the first 6 months of life, their immune system becomes strengthened. A strong immune system protects them from diseases that cause infant mortality [16]. With exclusive breast-breastfeeding (i.e. not mixing it with other feeds), the risk of HIV transmission is lowered [17].

The first breast milk (colostrum) gives the newborn natural protection from the mother to avert infections [18]. With exclusive breastfeeding for the first 6 months of life, babies achieve optimal growth and development [19]. Research has demonstrated that 6 months of EBF lowers the risk of transmission of HIV to the neonate by 3–4 times when compared to mixed breastfeeding [17] HIV status of the mother influences family choices on infant breastfeeding methods despite government policies such as maternal age, HIV stigma, education of the mother, economic factors, and cultural beliefs on breast milk [17, 20].

In the literature, the EBF among HIV infected mothers is affected by inadequate counselling on EBF practice [21, 22], cultural and family influence [21, 23], socio-economic factors [23],fear of stigmatization [22, 23], maternal lack of decision power [21] and fear of vertical transmission of HIV to infant [21].

Little was known about the prevalence and factors associated with exclusive breastfeeding in the southern highland of Tanzania, a region with a high prevalence of HIV-infected people. Therefore the study aimed at assessing the prevalence and associated factors of EBF among lactating HIV-infected mothers in the Southern Highland Zone of Tanzania.

## Method

### Study setting

The study was conducted in the Southern Highland Zone (Iringa and Njombe) regions. These regions are estimated to have the highest HIV prevalence within Tanzania, whereby 16.5% of adults are infected in Njombe and 19.2% in Iringa [24]. These two regions have therefore been selected because of the remarkably high prevalence of HIV. Iringa Region is served by a total of 36 health facilities, of which 13 are hospitals and 23 health centers. All these health facilities provide CTC and PMCTC services. The Iringa Region borders the dry belt of central Tanzania in the north and south by Lake Nyasa (Lake Malawi). It has six districts with a population of 941,238 based on the Tanzania national census 2012[24]. Njombe Region is among the 31 managerial regions of Tanzania. It was officially registered in March 2012, from the Iringa region as an independent region. The 2012 national census shows that the population of Njombe is 702,097 [24].

### Study design

A hospital-based analytical cross-sectional study employed a quantitative approach was used. The study population comprised HIV-positive lactating mothers attending the PMCT program in Iringa and Njombe Regions.

### Inclusion criteria

All HIV-positive lactating mothers with an infant aged 6 to 12 months who were attending the PMCT program during data collection were included.

### Exclusion criteria

All HIV-positive lactating mothers with very seriously ill children who were not able to concentrate on answering the questions were excluded from the study. Mothers diagnosed with cognitive or psychiatric conditions were also excluded as their level of comprehension would be limited. HIV-positive lactating mothers who were very seriously sick at the time of data collection were excluded as body weakness would result in the inability to not being able to go through all questions comprehensively.

### Sample size

The sample size was estimated by using the Kish Leslie formula (1965).

Formula:

$${\text{n}} = ( {\text{Z}} )^{2} {\text{P}}\left( 1 - {\text{P}} \right)/{\text{e}}^{2}$$

where, n = the required minimum sample size; Z = constant standard normal deviate (1.96% confidence level); P = estimated prevalence of HIV positive mothers who breastfeed exclusively up to 6 months of infants age which is 46% as per study by Saka [6]. **e** = margin of error on p (set at 5%)

Where by Z = 1.96; P = 46% = 0.46; e = 5% = 0.05; n = (1.96)^2^ × (0.46) (1–0.46)/(0.05)^2^ = 0.95**/**0.0025 = 372

Therefore, the actual sample size for this study was 372 HIV lactating mothers.

### Sampling technique

The Census method was used to include the regional hospitals; Iringa and Njombe Regional Hospitals. In other health facilities, a simple random procedure was employed. In Iringa, there are six districts. Out of these, 3 districts were selected by the lottery replacement method. Then one hospital was selected from each district using the lottery replacement method, and two health centers from each district. The same procedure of simple random method by lottery replacement was used in Njombe Region. Where by three districts out of six were selected; one hospital was selected from each district using the lottery replacement method, and two health centers from each district. Then, systematic random sampling was employed in which the first round of HIV-positive lactating mothers was identified from the clinic registration. In the second round, the calculation of the Kth interval was done using the Kth formula to select mothers who were invited to participate in the study.

$${\text{Kth}} = {\text{N}}/{\text{n}}$$

where by, N = total population units; n = sample size.

In this study, the total population of HIV lactating mothers was 963 and the sample size was 372.

### Data collection technique and tool

The data for this study was gathered through face-to-face interviewer-administered structured questionnaires adapted and modified from [6, 19, 20]. Data were collected by two trained research assistants and the principal investigator. Standard structured questionnaires were then translated by the language teacher to Kiswahili where the language is spoken by study participants. The Kiswahili version of the questionnaire was used.

### Variables and their measurements

#### Dependent variables

Exclusive breastfeeding (EBF) status was measured by the nominal scale. Exclusive breastfeeding practices were defined as continuous breastfeeding since birth such as starting to provide the baby with breast milk within the first hour after delivery, breastfeeding on demand day and night, and continuous breastfeeding alone for up to 6 months.

#### Independent variables

Socio-demographic characteristic comprised 19 questions and were measured by nominal scale i.e. residence, education, marital status, occupation, a model of delivery, place of delivery, counseling on EBF. Age and income were measured by an ordinal scale.

Knowledge about EBF was measured by a nominal scale involving 12 questions, with yes/no answers which were then converted into correct and incorrect. The total score was computed to categorize it into adequate and inadequate knowledge. The mean score of knowledge on EBF among lactating mothers was 7.66 the maximum score being 12 points while the minimum score was 1 point. A score below the mean was considered inadequate knowledge and above the mean adequate knowledge.

#### Perceived benefits of EBF

Perception of EBF was measured using a 5-point Likert scale. 13 questions were used to determine the perception of mothers. The total score was computed for the mean and then categorized into positive and negative perceptions. The mean score of perception on EBF among lactating mothers was 41.38 (of 60 points maximum) while the minimum score was 22 points. A score above the mean was regarded as a positive perception, while a score below the mean was considered a negative perception.

#### Data analysis

The descriptive statistics used were frequency and percentages in categorized variables like gender, infant HIV serostatus, areas of residence, education level, income level, marital status, and occupation. Frequency and percentages were used to determine the prevalence of outcome variables. The mean or median, standard deviations, and range were used to summarize continuous/discrete random variables such as age, and Likert scale items. In analyzing Likert scale items on perception and knowledge, the mean score was generated. The hypothesis was tested using chi-square to test the proportion of outcomes (EBF practices) across different exposures.

In building the logistic regression model, the process started from simple to complex analysis that led to parsimonious models. The independent variables were added one after another and those with significant results in the univariate were adjusted in the final model. The measure of effects was estimated by the Odds ratio and was tested at a 95% confidence interval and a 5% significance level.

## Results

### Social-demographic characteristics

This study included 372 breastfeeding women who had been diagnosed with HIV infection before or during pregnancy. Their mean age was 30.66 ± 5.72 (range 18–49) and that of their children at the time of data collection was 9.74 ± 2.08 (range 6–12 months). Most of the women were married 337 (90.6%), lived at or less than one USD per day 207(55.6%), and had completed only a primary level of education 199 (53.5%) (Table 1).

**Table 1:** Social-demographic distribution of the respondents (*N* = 372)

Variables	Frequency(n)	Percentages (%)
The age group of the mother		
Below 20 years	16	1.6
20–34 years	265	71.2
35–49 years	101	27.2
Education level		
No formal education	14	3.8
Primary	199	53.5
Secondary	141	37.9
College	18	4.8
Residence		
Rural	195	52.4
Urban	177	46.6
Occupation		
Employed	48	12.9
Self-employment	157	42.2
Housewife	167	44.9
The average income per day		
One Us dollar	207	55.6
Less than one dollar	165	44.4
Marital status		
Married	337	90.6
Unmarried	35	9.4
Sex of the child		
Female	200	53.8
Male	172	46.2
Place of delivery		
Hospital delivery	369	99.2
Home delivery	3	0.8
Mode of delivery		
Spontaneous vaginal delivery	305	82
Caesarean section	63	16.9
Assisted delivery	4	1.1
Counselled on EBF		
Counselled on EBF	371	99.7
Not counselled on EBF	1	0.3
Attended ANC		
Inadequate visit	138	37.1
Adequate visit	234	62.9

### Knowledge on EBF

The findings showed that 348 (96.2%) of respondents knew that breast milk was inexpensive and that the baby should be breastfed on demand 290 (78.0%). The majority of the women 352 (94.6%) felt that breastfeeding increases the bonding between the mother and the baby. Most of the respondents could define EBF (58%) and knew that colostrum is nutritious for infants (68%). Overall, there was good knowledge about the benefits of EBF for infants. In contrast, the majority did not know that EBF is a contraceptive method (lactational amenorrhea method) and that breastfeeding reduces the risk of maternal breast cancer (Table 2).

**Table 2:** Knowledge about EBF among lactating mothers with HIV (*N* = 372)

Knowledge on EBF	Incorrect *n* (%)	Correct *n* (%)
Is the early yellowish milk (colostrum) nutritious for the baby?	119 (32.0)	253 (68.0)
Do you think breast milk alone is sufficient for the baby for 0–6 months?	121 (32.5)	251 (67.5)
Should the baby be breastfed on demand?	82 (22.0)	290 (78.0)
The appropriate time to start complementary foods is soon as the baby starts the seventh month?	140 (37.6)	232 (62.4)
The child is supposed to be breastfed exclusively for 6 months	117 (31.5)	255 (68.5)
Does breast milk protect the baby from infection/diseases?	179 (48.1)	193 (51.9)
Does breast milk increase bonding between the mother and the baby?	20 (5.4)	352 (94.6)
Is it true that breast milk is cheap and available?	14 (3.8)	358 (96.2)
Exclusive breastfeeding act as a Contraception method?	232 (62.4)	140 (37.6)
Exclusive breastfeeding maintains mothers body weight	181 (48.7)	191 (51.3)
Exclusive breastfeeding prevents maternal breast cancer	253 (68.0)	119 (32.0)
Exclusive breastfeeding means give only breast milk to an infant for 6 months of life and medicines if indicated	156 (41.9)	216 (58.1)

The mean score for the perception of EBF among HIV lactating mothers was 41.38. The maximum score was 60 points while the minimum score was 22 points out of all respondents (N = 372), positive perceptions were 194(52.2%), and negative perceptions 178(47.8%).

### Prevalence of EBT practice

The prevalence of EBF practice was 58.1% at a 95% Confidence Interval of 52.9% to 63.1%. More than half of the respondents 216 (58.1%) had exclusively breastfed their infants for the first 6 months while 156 (41.9%) did not breastfeed their infants exclusively in the first 6 months.

### The relationship between lactating mothers’ characteristics and EBF practice

By cross-tabulation, there was a significant relationship between knowledge about EBF and the practice of EBF (p < 0.001) in which those who had adequate knowledge were most likely practicing EBF. Other variables that showed a significant relationship were the income of a mother (p = 0.002), level of education of a mother (p = 0.007), adequacy of antenatal visits (p < 0.001), perception of EBF (p < 0.001), and ever experienced breast problem (p < 0.001) refer to Table3.

**Table 3:** The relationship between knowledge on EBF and EBF practice

Variables	EBF f (%)	No EBF f(%)	*Χ*^2^	*p*-value
The age group of the mother				
Below 20 years	4(66.7)	2 (33.3)		
20–34 years	157(59.2)	108 (40.8)		
35–49 years	55(54.5)	46(45.5)	0.87	0.646
Sex of the child				
Male	104(60.5)	68(39.5)		
Female	112(56.0)	88(44.0)	0.76	0.384
Income per day				
1USD	135(65.2)	72(34.8)		
< 1USD	81(49.1)	84(50.9)	9.81	0.002
Level of education				
No formal education	4(28.6)	10(71.4)		
Primary	106(53.3)	93(46.7)		
Secondary	95(67.4)	46(32.6)		
College	11(61.1)	7(38.9)	11.97	0.007
Occupation				
Employed	30(65.5)	18(37.5)		
Self-employed	96(61.1)	61(38.9)		
Housewife	90(53.9)	77(46.1)	2.19	0.334
Residence				
Rural	108(55.4)	87(44.6)		
Urban	108(61.0)	69(39.0)	1.21	0.272
Mode of delivery				
Normal delivery	181(53.3)	124(40.7)		
Cesarean section	34(54)	29(46)		
Assisted delivery	1(25)	3(75)	2.44	0.296
Counseled on EBF				
Yes	216(58.2)	155(41.8)		
No	0(0.0)	1(100)	1.39	0.239
ANC VISIT				
Inadequate visit	64(46.4)	74(53.6)		
Adequate visit	152(65.0)	82(35.0)	12.31	< 0.001
Knowledge categories				
Inadequate knowledge	64(37.0)	109(63.0)		
Adequate knowledge	152(76.4)	47(23.6)	58.96	< 0.001
Perception of EBF				
Negative	102(57.3%)	76(42.7%)		
Positive	54(27.8%)	140(72.2%)	33.11	< 0.001
Experienced breast problem				
Yes	1			
No	3.595	1.876	6.89	< 0.001

### Factors associated with EBF practice among lactating women with HIV

After adjusting for confounders, factors associated with EBF practice among lactating HIV infected mothers were knowledge about EBF [Adequate knowledge (AOR = 5.11 at 95% CI 3.2–8.17, p < 0.001)], ANC visit [Adequate (AOR = 1.76 at 95% CI 1.09–2.82, p = 0.002)], Income per day [1 0r more USD (AOR = 1.83 at 95% CI 1.14–2.94, p = 0.013)], perception towards EBF [positive perception (AOR = 3.51 at 95% CI 2.25–5.47, p < 0.001) and ever experienced breast problem AOR = 3.91 at 95% CI 1.89–8.08, p < 0.001 (Table 4).

**Table 4:** The association between knowledge about EBF and practice

Variables	OR	95% CI		*p*-value	AOR	95% CI		*p*-value
		Lower	Upper			Lower	Upper	
ANC visits								
Inadequate	1				1			
Adequate	2.14	1.40	3.29	< 0.001	1.76	1.09	2.82	0.002
Knowledge on EBF								
Inadequate	1				1			
Adequate	5.51	3.51	8.64	< 0.001	5.11	3.20	8.17	< 0.001
Income per day								
Less than 1USD	1				1			
1 or more USD	1.94	1.28	2.95	0.002	1.83	1.14	2.94	0.013
Level of education								
No formal education	1				1			
Primary	2.85	0.87	9.39	0.085	2.33	0.62	8.74	0.210
Secondary	5.16	1.54	17.35	0.008	3.17	0.83	12.14	0.093
College	3.93	0.88	17.56	0.073	1.40	0.27	7.38	0.691
Perceptions of EBF								
Negative	1				1			
Positive	3.48	2.26	3.36	< 0.001	3.51	2.25	5.47	< 0.001
Experienced breast problem								
Yes	1				1			
No	3.60	1.88	6.89	< 0.001	3.91	1.89	8.08	< 0.001

## Discussion

More than half (58%) of the interviewed women reported practicing EBF. The prevalence was found to be almost the same as the national average of EBF in the general population of Tanzania [14]. This is a worrisome situation because mixed feeding in the first 6 months of life is associated with an increased risk of vertical transmission of HIV/AIDS [25]. The finding was higher compared to previous studies done among women infected by HIV in Ilala Municipal Dar es Salaam [13] and Ethiopia [26]. The lower EBF practice in the previous study could be due to differences in the socioeconomic status of the two-study population. HIV-infected mothers in the city could have opted for an alternative nutritional diet to replace breastfeeding if compared to HIV-infected mothers in the current study population.

The current study revealed that slightly more than half of the respondents had adequate knowledge about exclusive breastfeeding. This is lower than what has been reported in other studies in Africa, including a study done in South Africa where more than three-quarters of the respondents were knowledgeable about EBF [27]. It is evidenced that knowledge of EBF is significantly associated with uptake of exclusive breastfeeding [28].

This study demonstrated that respondents with adequate knowledge of EBF practice were nearly two times more likely to practice EBF than their counterparts with inadequate knowledge. This is consistent with Ethiopian and South African studies which reported that knowledgeable respondents were five times more likely to practice EBF [29, 30]. Knowledge informs practice at the community level. This demonstrates an urgent need to empower expectant women and their families with knowledge about the many benefits of EBF. An important source of health information is derived from routine prenatal visits (ANC) and in our study, adequate ANC attendance increased the odds of EBF practice, especially among pregnant women who attended ANC services more than 4 times. Most pregnant people rely on ANC visits for more information about maternal and child health. This association between attendance to ANC, level of knowledge, and the practice of EBF have also been reported by Alebel et al. [30] in Ethiopia. The study recommends innovative strategies to strengthen community education that would be a worthwhile investment in support of maternal and child health, especially among women living with HIV.

In this current study more than half of the respondents had a positive perception of exclusive breasting feeding. Interestingly, a similar study done in South Africa reported that the majority of the respondents had a positive perception of EBF [27]. The difference in perception could be attributed to the difference in the level of knowledge of EBF between the two settings. There are also sociocultural and belief differences that may affect how EBF is perceived from one setting to another. It is important to create a positive belief/perception as shown in the health belief model that this is what ignites the process of change of behavior [31].

This is also evident in the current study where it is found that respondents with positive perceptions were four times more likely to practice EBF than those who had a negative perception. The finding of the present study was consistent with the study conducted in Ethiopia by Gebeyehu et al. [29] which noted that respondents who had positive perceptions were seven times more likely to practice EBF. Positive perception could be encouraged through counseling on infant feeding practice during ANC but also by showing for example the consequences of not breastfeeding exclusively [29]. Again, following the Health Belief Model, pregnant and lactating women will most likely choose EBF if they are convinced that this is possible even within limited time and resources and if they understand the benefit for their infant.

The present results revealed that the majority of respondents identified insufficient breast milk as a barrier to practicing EBF. The finding was similar to the study done by Maonga et al. [32] in Muheza Tanzania which reported that insufficient breast milk was one of the hindrances to practicing EBF. However, the finding from these two Tanzanian studies is different from a report from South Africa showing that the most prominent barriers to EBF were cultural factors and influence from elders in the family [30]. In Kenya, stigma was the most common barrier to EBF among HIV-infected lactating mothers [33].

The study further indicated that respondents who did not experience breast problems were three times more likely to practice EBF than their counterparts with breast problems. Breast problems were also identified as a barrier to EBF in another study in Nigeria [34]. Screening for breast conditions during pregnancy and lactation is therefore of paramount importance so that treatable conditions can be identified and given due management. This will likely increase the prevalence of EBF and improve infants' health.

In another study by Muhammed & Seid [20], it was found that HIV –infected mothers who were formally employed were ten times more likely to be non-exclusive breast-feeders than the unemployed. The current study did not find this a significant barrier. These differences could be due to educational status as with the current study the majority had a primary level of education and the study subjects did not differ so much in employment status. However, with the growing pace of women's education and the acquisition of full-time jobs, the means through which EBF can be promoted in this population ought to be considered.

This study was not without limitations, being cross-sectional it cannot establish a causal effect relationship. It sets a basis for an interventional study by establishing the factors influencing exclusive breastfeeding among HIV-infected lactating mothers.

## Conclusion

More than half of interviewed mothers with HIV practiced EBF. EBF practice was significantly influenced by adequate knowledge about EBF, positive perception about EBF, adequate number of ANC visits and having never experienced breast problems. Strengthening adherence to ANC routine visits, counseling on breastfeeding, and improving mothers' knowledge about exclusive breastfeeding would contribute to the enhancement of EBF practice in this region. An innovative interventional study is recommended to come up with effective strategies to improve the overall health of infants through EBF practice among HIV-infected mothers.

## Data Availability

The data and material used in the current study are available from the corresponding authors upon request.

## Abbreviations

ANC: Antenatal clinic
ARV: Antiretroviral medictions
CTC: Care and treatment centre for HIV
EBF: Exclusive breastfeeding
HBM: Health belief model
HIV: Human immunodeficiency virus
ID: Identification
MSc: Masters of science
MTCT: Mother to child transmission
ORS: Oral rehydration salt
PMTCT: Prevention of mother to child transmission
RCH: Reproductive child health
SDGs: Sustainable development goals
UDOM: University of Dodoma
UNAIDS: United Nations Programme on HIV/AIDS
WHO: World Health Organization
